# Long noncoding RNA NR2F1-AS1 plays a carcinogenic role in gastric cancer by recruiting transcriptional factor SPI1 to upregulate ST8SIA1 expression

**DOI:** 10.1080/21655979.2021.2001168

**Published:** 2021-12-13

**Authors:** Fang Zuo, Yong Zhang, Jianting Li, Shaoxiang Yang, Xiaolu Chen

**Affiliations:** a Department of Health Care, Jinan Central Hospital, Jinan, Shandong, China; b Department of Spleen and Stomach Diseases, Liaocheng Chinese Medicine Hospital, Liaocheng, Shandong, China; c Department of Oncology, Jinan Central Hospital, Jinan, Shandong, China

**Keywords:** Gastric cancer, LncRNA NR2F1-AS, SPI1, ST8SIA1

## Abstract

Gastric cancer (GC) is a highly malignant solid tumor of the digestive tract, which is associated with a high mortality rate. Long non-coding RNA (lncRNA) nuclear receptor subfamily 2 group F member 1 antisense RNA 1 (NR2F1-AS1) has been reported to exert a tumor-promoting effect in some types of cancer. The present study aimed to investigate the role of NR2F1-AS1 in GC. The expression levels of NR2F1-AS1 and its potential target gene were measured in GC cell lines. Bioinformatics analysis, an RNA immunoprecipitation assay and a chromatin immunoprecipitation assay were used to determine the binding relationship between NR2F1-AS1 and downstream genes. The effect of NR2F1-AS1 regulatory axis on AGC cell viability, proliferation, migration, invasion and epithelial-mesenchymal transition was evaluated. The results of the present study revealed that the knockdown of NR2F1-AS1 inhibited the proliferation, invasion and migration of GC cells. NR2F1-AS1 also upregulated the expression levels of ST8SIA1 by recruiting transcriptional factor SPI1. Thus, the effects of the knockdown of NR2F1-AS1 on GC cell functions were suggested to occur via regulation of ST8SIA1. In conclusion, the findings of the current study indicated that NR2F1-AS1 may promote the proliferation, invasion and migration of GC cells by recruiting SPI1, to upregulate ST8SIA1 expression. Thus, the regulation of their expression levels may provide a novel direction for the treatment of GC.

## Introduction

Gastric cancer (GC) is one of the malignant tumors of digestive tract with high degree of malignancy and accounts for some of the highest rates of cancer-associated high morbidity and mortality globally [1,2]. In fact, GC is the second most common cause of cancer-related mortality [3]. GC risk is considered to be closely related to gastritis, and the long-term consumption of pickles containing nitrite, alcohol, smoking and high-salt diets, are all risk factors for GC [4]. Thus, the risk factors for GC seem to be ubiquitous in daily habits. On account of patients with early GC without obvious clinical symptoms, it is often difficult to identify and diagnose early stage GC. Therefore, due to the lack of effective early detection and diagnosis methods and effective screening procedures, most cases were diagnosed as advanced GC with poor prognosis [5]. Currently, the treatment regimen of GC mainly comprises surgery, chemotherapy, and radiotherapy according to the different stages. Although the 5-year survival rate of patients has been improved, the early diagnosis rate is not optimistic [6]. Therefore, there remains an urgent requirement to discover novel and preferred noninvasive biomarkers for the early diagnosis and detection of GC.

It has been demonstrated that long noncoding RNAs (lncRNAs) play a crucial role in regulating numerous biological functions, and promote the development of various types of cancer, including bladder cancer [7], GC [8], papillary thyroid cancer [9], and retinoblastoma [10]. For example, one study reported that lncRNA nuclear receptor subfamily 2 group F member 1 antisense RNA 1 (NR2F1-AS1) promoted angiogenesis in breast cancer by activating the insulin-like growth factor 1 (IGF-1)/IGF-1 receptor/ERK signaling pathway [11]. In another study, NR2F1-AS1 increases the expression of forkhead box A1 through microRNA (miR)-483-3p sponges, which enhances the malignant degree of osteosarcoma [12]. NR2F1-AS1 also regulated miR-423-5p/SRY-box transcription factor 12 (SOX12) axis to promote the proliferation and invasion of thyroid papillary carcinoma cells [13]. Furthermore, NR2F1-induced NR2F1-AS1 was discovered to promote the progression of esophageal squamous cell carcinoma by activating the Hedgehog signaling pathway [14]. NR2F1-AS1 was also found to promote the proliferation and migration of thyroid cancer cells and inhibits apoptosis by regulating the microRNA-338-3P/CCND1 axis [15]. However, to the best of our knowledge, studies investigating the role of NR2F1-AS1 in GC has yet to be reported.

The present study aimed to unveil the association between NR2F1-AS1 and its target genes. In addition, the study sought to investigate the role of the NR2F1-AS1 regulatory axis in proliferation, invasion and migration of GC cells, which may provide a novel insight into potential therapeutic strategies for GC.

## Materials and methods

### Cell culture and cell transfection

The human gastric epithelial cell line GES-1 and GC cell lines (KKP, KE-39, AGS and MKN-45) were purchased from BeNa Culture Collection (BNCC, Beijing, China) and cultured in the DMEM medium (Gibco; Thermo Fisher Scientific) supplemented with 10% FBS (Gibco; Thermo Fisher Scientific) under constant conditions at 37°C in a humidified atmosphere with 5% CO_2_.

Knockdown was performed with pLKO.1 shRNAs against NR2F1-AS1 (shRNA-NR2F1-AS1#1 and shRNA-NR2F1-AS1#2), shRNAs against Spi-1 proto-oncogene (SPI1) (shRNA- SPI1#1 and shRNA-SPI1#2), and non-targeted shRNA as negative control (shRNA-NC). shRNA#1 and shRNA#2 correspond to different sequences being incorporated into the same vector. They were purchased from VectorBuilder Biotechnology (Guangzhou, China). The pcDNA3.1 vector overexpressing NR2F1-AS1 (Oe-NR2F1-AS1), ST8 alpha-N-acetyl-neuraminide alpha-2, 8-sialyltransferase 1 (ST8SIA1) (Oe-ST8SIA1) and the empty vector as negative control (Oe-NC) were constructed by Fenghui Biotechnology (Changsha, China). Cell transfection was performed according to manufacturer’s instruction of Lipofectamine^TM^ 3000 (Invitrogen).

### Cell proliferation assay

MTT and colony formation assays were used to evaluate the ability of cell proliferation, as previously described [16]. In brief, AGS cells (1 × 10^3^ cells/per well) were seeded in 96-well plates and cultured for 24, 48 and 72 h. After the incubation, MTT solution (Sigma, St. Louis, Missouri, USA) was added to each well. The cells were incubated for an additional 1 h to determine the cell viability. The optical density was determined at 450 nm using a microplate reader.

AGS cells were seeded into 60 mm-cell dish. After an incubation period of 14 days at 37°C, the colonies were fixed with 100% methanol and stained with 0.1% crystal violet in absolute ethanol for 15 min. Visible colonies of more than 50 cells were visualized and analyzed using Image J v1.46 software (National Institutes of Health).

### Wound healing and transwell assays

For the wound-healing assay, transfected AGS cells were seeded into six-well plates and cultured to 70–80% confluence. The cell monolayer was then scratched with a 200-μl sterile pipette tip to create an artificial wound. After washing with PBS, the cells were cultured in serum-free medium. Images were acquired at each time point (0 and 24 h).

For Transwell assay, the cell invasive ability was evaluated using Transwell chambers, in which the upper chamber was precoated with Matrigel (BD Biosciences). In brief, AGS cells (3 × 10^4^ cells) were plated in the serum-free medium into the upper chamber as a 0.1 ml cell suspension. The lower chamber was filled with cell culture medium containing 20% FBS. Following 24 h of incubation, the invasive cells in the lower chamber were stained with 0.5% crystal violet (Sigma-Aldrich) at room temperature for 10 min. Stained cells were visualized under a light microscope.

### Dual luciferase reporter assay

ST8SIA1 fragments containing the wild-type (WT) NR2F1-AS1 binding sites or its mutated form (MUT) were inserted downstream of the firefly luciferase gene in the pGL3 vector (Promega), and the constructed vectors were denoted as pGL3-ST8SIA1-WT and pGL3-ST8SIA1-MUT, respectively. For the luciferase reporter assay, AGS cells were seeded into 24-well plates and cultured for 24 h. Then, cells were co-transfected with shRNA-NR2F1-AS1 or shRNA-NC and 1000 ng of ST8SIA1- WT or – MUT vector using Lipofectamine™ 3000 (Invitrogen). The luciferase activity was determined by a Dual-Luciferase Reporter Assay System (E1910, Promega) according to the manufacturer’s recommendations and normalized to *Renilla* luciferase activity. The relative luciferase activity is expressed as a ratio of firefly/Renilla luciferase activity.

### RNA immunoprecipitation (RIP)

The ability of NR2F1-AS1 to bind SPI1 was detected using the RIP kit (Millipore Sigma, Burlington, MA, USA). The cells were lysed with an equal volume of RIPA lysis (P0013B, Beyotime Biotechnology) in an ice bath for 5 min. Part of the cell extract was saved as the input, and the remaining was incubated with anti-IgG (1: 100, ab109489, Abcam) or anti-SPI1 (1: 1000, sc-365,208, Santa Cruz Biotechnology Inc.) antibodies for co-precipitation. The bead-protein complex was collected on a magnetic base. RNA was extracted from the co-precipitated samples and Input samples after detachment from the bead using protease K. The expression of NR2F1-AS1 was determined using RT-qPCR.

### RNA pull down assay

The ability of NR2F1-AS1 to bind to SPI1 was also determined using a Pierce^TM^ Magnetic RNA-Protein Pull-Down kit (cat. no. 20164; Thermo Fisher Scientific, Inc.) according to the manufacturer’s instructions. Briefly, RNA was first labeled using Pierce^TM^ RNA 3ʹEnd Desthiobiotinylation kit (cat. no. 20163; Thermo Fisher Scientific, Inc.). AGS cells lysate was obtained by processing cells as in the RIP assay. Following resuspending the magnetic beads, labeled-RNA was added and co-incubated at room temperature for 30 min. And beads were collected by a magnetic force followed by the addition of cell lysate, and they were rotated slowly at 4°C for 2 h. Finally, the elution buffer and the magnetic beads were vortexed and incubated at 37°C for 20 min. The supernatant was collected for detection by Western blotting.

### Chromatin immunoprecipitation (ChIP) assay

The ability of SPI1 to bind to ST8SIA1 was detected by a ChIP assay. The cells were fixed with 16% methanol and cross-linked, then treated with lysis buffer and sonicated. The cells were subsequently incubated with an anti-SPI1 antibody (1: 1000, sc-365,208, Santa Cruz Biotechnology Inc.) overnight. Next, beads were added to harvest the protein-DNA complex. Then decrosslinking was performed with addition of 5 mmol/l NaCl to retrieve DNA. The enrichment of SPI1was examined using RT-qPCR.

### Western blotting

Total protein was extracted from AGS cells using RIPA lysis buffer and quantified by a Pierce BCA Protein assay kit (Thermo Fisher Scientific). After denaturing, proteins were separated via 12% SDS-PAGE and the separated proteins were subsequently transferred onto PVDF membranes, which were blocked with 5% fat-free milk for 2 h at room temperature. After washed, membranes were incubated with primary antibodies at 4°C overnight and subsequently incubated with an IgG-HRP-conjugated goat anti-rabbit secondary antibody for 1 h at room temperature. Protein bands were visualized using ECL reagent (Millipore). Densitometric analysis were performed using Image J v1.46 software. Information about antibodies are as follows: E-cadherin (ab40772; 1:10,000), N-cadherin (ab245117; 1:1,000), Vimentin (ab92547; 1,000), snail family transcriptional repressor 1 (Snail; ab216347; 1,000), ST8SIA1 (ab253021; 1:300), SPI1 (ab227835; 1:1,000), secondary antibody (ab6721; 1:5,000; all Abcam).

### Bioinformatics and statistical analysis

The possible underlying mechanism of NR2F1-AS1 in GC was predicted using the lncRNA Modulator Atlas in Pan-cancer (LNCMAP) database (http://www.bio-bigdata.com/LncMAP).

All experiments were repeated at least 3 times independently and results are expressed as mean ± standard deviation (SD). Statistical analyses were performed using SPSS 19.0 software (SPSS, Chicago, IL, USA). Student’s t-test or one-way ANOVA followed by Tukey’s post hoc test were used to evaluate the statistical significance. P < 0.05 was considered to be statistically significant.

## Results

The present study aimed to investigate the role of NR2F1-AS1 in GC. The results of the present study revealed that the knockdown of NR2F1-AS1 inhibited the proliferation, invasion and migration of GC cells. NR2F1-AS1 also upregulated the expression levels of ST8SIA1 by recruiting SPI1. Thus, the effects of the knockdown of NR2F1-AS1 on GC cell functions were suggested to occur via regulation of ST8SIA1. In conclusion, the findings of the current study indicated that NR2F1-AS1 may promote the malignant progression of GC cells by recruiting the transcriptional factor, SPI1, to upregulate ST8SIA1.

### Knockdown of lncRNA NR2F1-AS1 suppressed the proliferation, invasion and migration of GC cells

**Figure 1.**
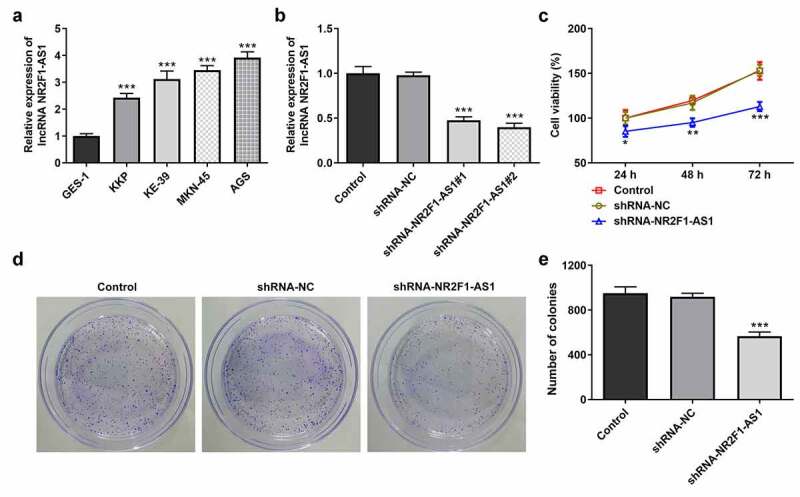
Knockdown of long non-coding RNA NR2F1-AS1 suppresses the viability and proliferation of gastric cancer cells. (a) Relative mRNA expression of NR2F1-AS1 in different cell lines was measured using RT-qPCR. ***P < 0.001 vs. GES-1. (b) Relative mRNA expression of NR2F1-AS1 in the control and transfection group was detected using RT-qPCR. ***P < 0.001 vs. shRNA. (c) MTT and (d and e) colony formation assays were performed to determine the viability and proliferation of NR2F1-AS1-silenced AGS cells. *P < 0.05, **P < 0.01, ***P < 0.001 vs. shRNA-NC

**Figure 2.**
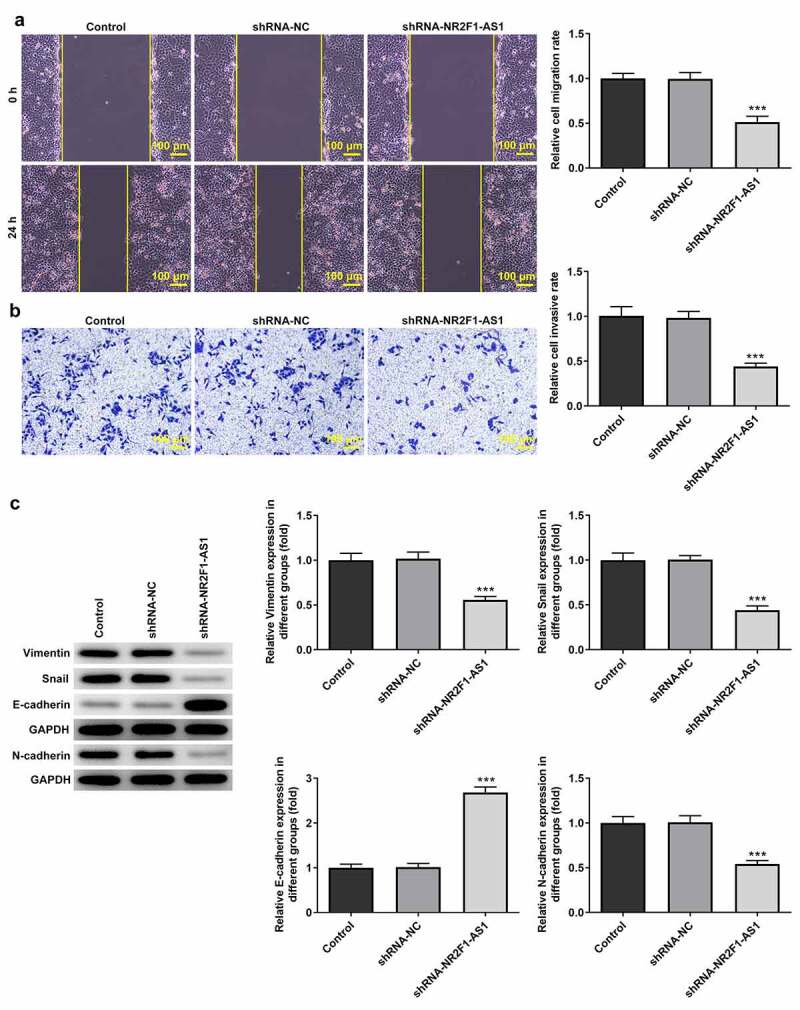
Knockdown of long non-coding RNA NR2F1-AS1 suppresses the invasion and migration of gastric cancer cells. (a) Relative migration rate was detected using a wound healing assay. (b) Relative cell invasion rate was detected using a Transwell assay. (c) Expression levels of epithelial-mesenchymal transition-related proteins were measured using Western blotting. ***P < 0.001 vs. control or shRNA-NC

The results of the RT-qPCR experiments revealed that the expression levels of NR2F1-AS1 were upregulated in GC cells (KKP, KE-39, MKN-45 and AGS) compared with those in GES-1 cells (Figure 1a). The expression levels of NR2F1-AS1 were upregulated to the greatest extent in AGS cells; thus, this cell line was selected for use in subsequent experiments. The role of NR2F1-AS1 in GC cells was further explored by transfecting shRNA-NR2F1-AS1 into AGS cells. The expression of NR2F-AS1 after transfection was detected using RT-qPCR, and the results revealed that NR2F1-AS1 expression levels were downregulated in AGS cells transfected with shRNA-NR2F1-AS1#1 or shRNA-NR2F1-AS1#2. shRNA-NR2F1-AS1#2 was selected for use in subsequent experiments, as it was able to downregulate NR2F1-AS1 expression to the greatest extent (Figure 1b). The results of the MTT (Figure 1c) and colony formation (Figure 1d and e) assays demonstrated that cell proliferation was decreased in AGS cells following the knockdown of NR2F-AS1 compared with that in the shRNA-NC and control groups. As shown in Figure 2a and b, the knockdown of NR2F-AS1 significantly decreased the migration rate of AGS cells. Taken together, these results suggested that the knockdown of NR2F1-AS1 may suppress the proliferation, invasion and migration of GC cells. Additionally, since epithelial-mesenchymal transition (EMT) is a vital effect of cancer metastasis [17], the expression levels of EMT-related proteins were detected by Western blot. As demonstrated in Figure 2c, NR2F1-AS1 inhibition significantly upregulated the expression levels of E-cadherin and downregulated the expression levels of N-cadherin, Snail and vimentin.

### NR2F1-AS1 upregulates ST8SIA1 expression by recruiting SPI1

**Figure 3.**
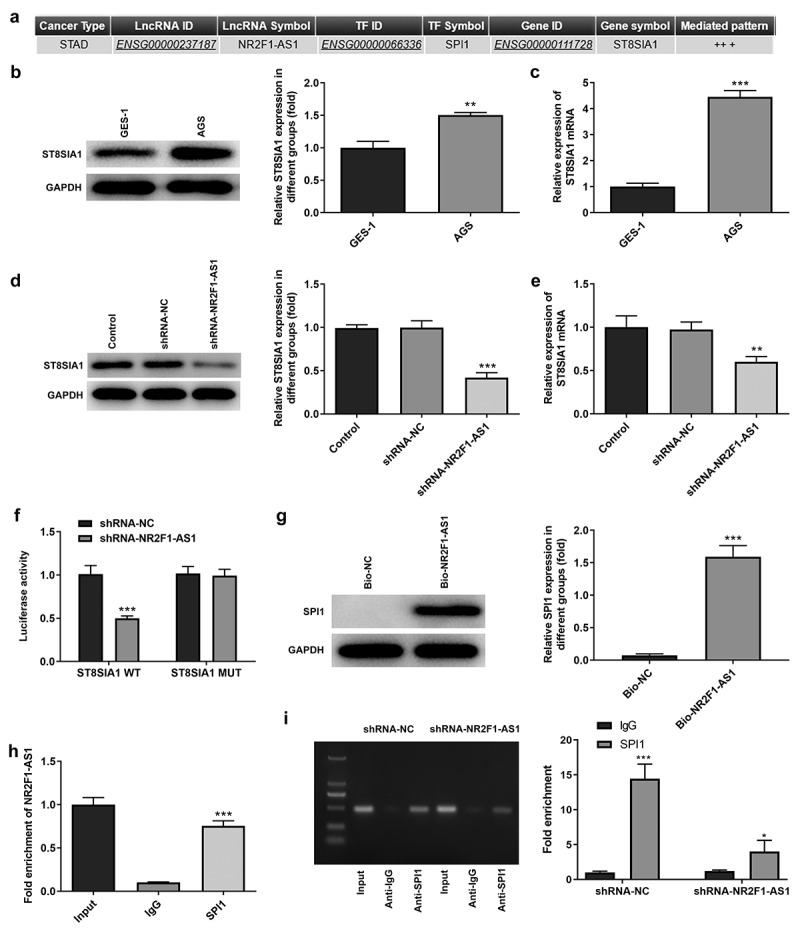
NR2F1-AS1 upregulates ST8SIA1 expression by recruiting SPI1. (a) lncRNA Modulator Atlas in Pan-cancer database was used to determine that NR2F1-AS1 bound with ST8SIA1 by recruiting SPI1. Relative (b) protein and (c) mRNA expression levels of ST8SIA1 in GES-1 and AGS cells were detected using Western blotting and RT-qPCR, respectively. Relative (d) protein and (e) mRNA expression levels were detected of ST8SIA1 in the control and transfection group using Western blotting and RT-qPCR, respectively. (f) shRNA-NR2F1-AS1 co-transfected with the pGL3-ST8SIA1-WT/MUT luciferase reporter was used to confirm the binding relationship between NR2F1-AS1 and ST8SIA1. (g) RNA pull-down and (h) RNA immunoprecipitation assays were performed to detect the binding relationship between NR2F1-AS1 and SPI1. (i) Chromatin immunoprecipitation assay was performed to further verify the binding between ST8SIA1 and SPI1. *P < 0.05, **P < 0.01, ***P < 0.001 vs. GES-1, shRNA-NC or IgG

The possible underlying mechanism of NR2F1-AS1 in GC was predicted using LNCMAP database (Figure 3a). The results of the RT-qPCR and Western blotting experiments revealed that ST8SIA1 expression was markedly upregulated in AGS cells compared with that in GES-1 cells (Figure 3b and c). In addition, RT-qPCR and Western blotting results demonstrated that the expression levels of ST8SIA1 were downregulated in the shRNA-NR2F1-AS1 group (Figure 3d and e). The results of the dual luciferase reporter assay discovered that the relative luciferase activity was not significantly different between the pGL3-ST8SIA2-MUT groups, whereas the relative luciferase activity was significantly decreased in the shRNA-NR2F1-AS1 group compared with the shRNA-NC group when co-transfected with the pGL3-ST8SIA2-WT vector, indicating that NR2F1-AS1 may affect the activity of the ST8SIA1 promoter (Figure 3f). RNA pull-down (Figure 3g) and RIP (Figure 3h) assays were subsequently performed to detect the relationship between NR2F1-AS1 and SPI1. Through determining the expression levels of the RNA-protein complex, the high expression levels of SPI1 observed in the RNA pull-down assay and those of NR2F1-AS1 in the RIP assay suggested that SPI1 may bind with NR2F1-AS1. In addition, a ChIP assay was also performed to detect the relationship between SPI1 and ST8SIA1. The results revealed that the enrichment of SPI1 in the shRNA-NR2F1-AS1 group was significantly decreased compared with that in the shRNA-NC group, suggesting that SPI1 could bind to ST8SIA1 (Figure 3i). These findings verified the relationship between the three components in the NR2F1-AS1/SPI1/ST8SIA1 regulatory axis.

### Knockdown of NR2F1-AS1 suppresses the proliferation, invasion and migration of GC cells by regulating ST8SIA1

**Figure 4.**
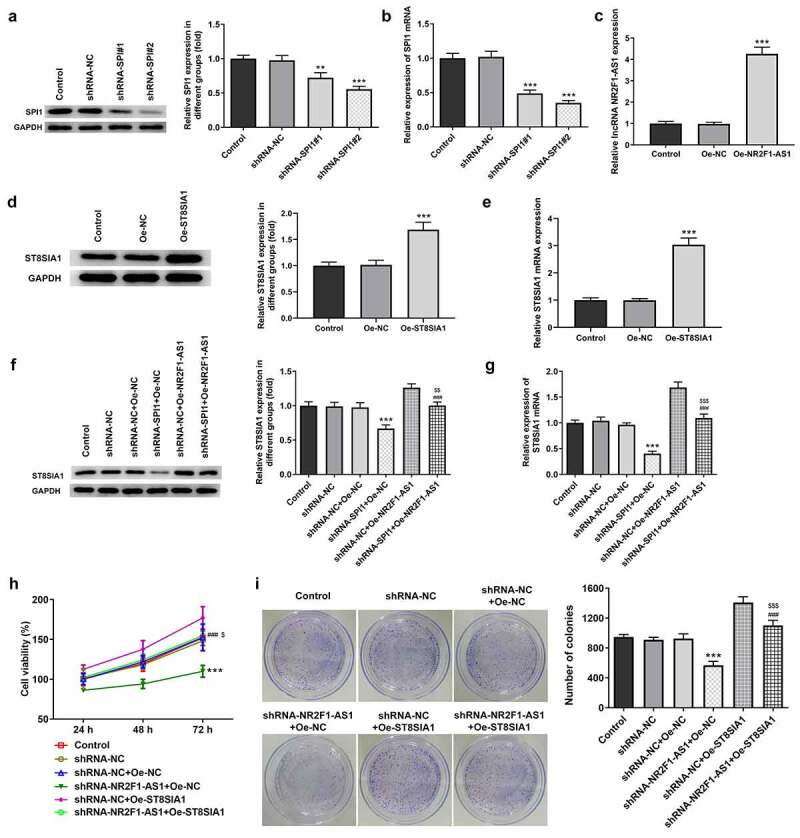
Knockdown of NR2F1-AS1 suppressed proliferation of gastric cancer cells through ST8SIA1. Relative (a) protein and (b) mRNA expression levels in the control and SPI1 knockdown groups were detected using Western blotting and RT-qPCR, respectively. (c) Relative mRNA expression levels in the control and Oe-NR2F1-AS1 group were detected using RT-qPCR. Relative (d) protein and (e) mRNA expression levels in the control and Oe-ST8SIA1 group were detected using Western blotting and RT-qPCR, respectively. Relative (f) protein and (g) mRNA expression levels in the control and co-transfection group were detected using Western blotting and RT-qPCR, respectively. (h) MTT and (i) colony formation assays were used to analyze the viability and proliferation of NR2F1-AS1-silenced and ST8SIA1-OeAGS cells. **P < 0.01, ***P < 0.001 vs. shRNA-NC; ^###^P < 0.001 vs. shRNA-NR2F1-AS1 + Oe-NC; ^$$^P < 0.01, ^$$$^P < 0.001 vs. shRNA-NC + Oe-ST8SIA1

To determine the effect of NR2F1-AS1 on the proliferation, invasion and migration of GC cells, the transfection efficiency of shRNA-SPI1, oe-NR2F1-AS1 and oe-ST8SIA1 in AGS cells was measured. The results demonstrated that the expression levels of SPI1 were downregulated in the knockdown groups (Figure 4a and b). As shRNA-SPI1#2 exhibited the most significant knockdown effect, it was selected for use in subsequent experiments. In addition, the expression levels of NR2F1-AS1 (Figure 4c) and ST8SIA1 (Figure 4d and e) were significantly upregulated in the overexpression groups. As shown in Figure 4f and g, the expression levels of ST8SIA1 were downregulated in shRNA-SPI1-transfected GC cells and upregulated in NR2F1-AS1-overexpressing GC cells. ST8SIA1 overexpression reversed the effects of SPI1 silencing on the expression levels of ST8SIA1 in GC cells.

In addition, as shown in Figure 4h and i, the decreased rate of cell proliferation induced by NR2F1-AS1 knockdown was partially increased by Oe-ST8SIA1, suggesting that the overexpression of ST8SIA1 may partly abolish the inhibitory effects of NR2F1-AS silencing on GC cell proliferation.

**Figure 5.**
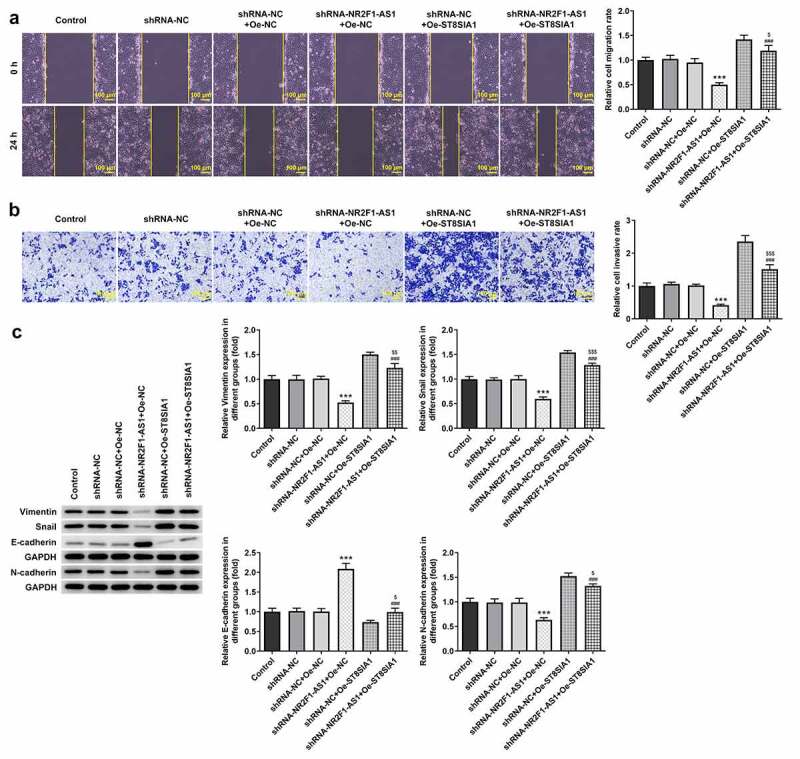
Knockdown of NR2F1-AS1 suppresses the invasion and migration of gastric cancer cells by regulating ST8SIA1. (a) Relative migration rate was detected using a wound healing assay. (b) Relative cell invasion rate was detected using a Transwell assay. (c) Expression levels of epithelial-mesenchymal transition-related proteins were measured using Western blotting. ***P < 0.001 vs. shRNA-NC; ^###^P < 0.001 vs. shRNA-NR2F1-AS1 + Oe-NC; ^$^P < 0.05, ^$$^P < 0.01, ^$$$^P < 0.001 vs. shRNA-NC + Oe-ST8SIA1

Compared with the shRNA-NR2F1-AS1 + Oe-NC group, the decreased migratory and invasive rates of AGS cells were subsequently increased following the transfection with Oe-ST8SIA1 (Figure 5a and b). Moreover, the expression levels of E-cadherin, N-cadherin, vimentin and Snail were detected using Western blotting, and all protein except E-cadherin were found to be downregulated in the shRNA-NR2F1-AS1 + Oe-ST8SIA1 group compared with those in the shRNA-NR2F1-AS1 + Oe-NC group (Figure 5c). In addition, as shown in Figure 5c, ST8SIA1 overexpression partly downregulated the expression levels of E-cadherin and upregulated the expression levels of N-cadherin, vimentin and Snail compared with those in the shRNA-NR2F1-AS1 + Oe-NC group.

## Discussion

GC, which poses a significant threat to human health, is one of the most aggressive and fatal types of cancer worldwide [5,18]. To date, the treatment of GC has been challenging and relatively limited, and more effective methods than the current treatments available remain to be determined [19]. A large number of studies have shown that lncRNAs are extensively involved in the biological processes of cancer cells and other cells [16,20,21]. A previous study found that NR2F1-AS1 expression was significantly upregulated in oxaliplatin-resistant hepatocellular carcinoma tissues [22]. Another study has shown that NR2F1-AS1 was highly expressed in hepatocellular carcinoma and its expression was found highly correlated with staging [22]. Moreover, Li et al found that the NR2F1-AS1/miR-140/ hexokinase 2 axis regulated hypoxia-induced glycolysis and migration in hepatocellular carcinoma [23]. In addition to hepatocellular carcinoma, NR2F1-AS1 was also demonstrated to regulate the miR-423-5p/SOX12 axis to promote the proliferation and invasion of papillary thyroid carcinoma cells [13]. Moreover, NR2F1-AS1 was also found to promote the proliferation and migration of thyroid cancer cells and inhibited apoptosis [15]. These results suggested that NR2F1-AS1 may affect the proliferation, invasion and apoptosis of the aforementioned cancer cells. In the present study, the results revealed that NR2F1-AS1 expression levels were upregulated in GC cells. Furthermore, the knockdown of NR2F1-AS1 expression could suppress the proliferation, migration and invasiveness of AGS cells.

**Figure 6.**
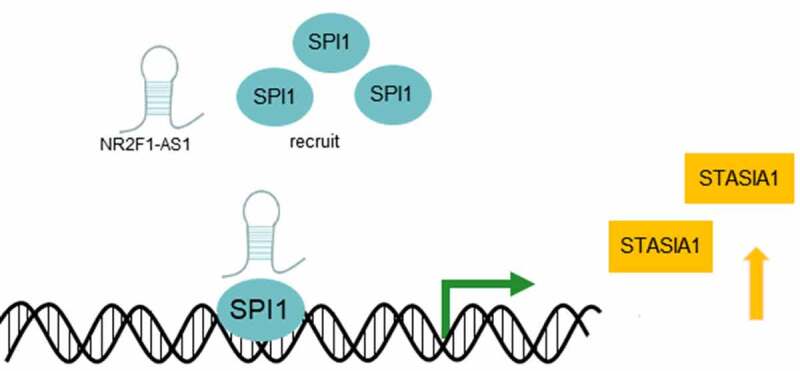
NR2F1-AS1 upregulates the expression levels of ST8SIA1 by recruiting SPI1

The current study also used the LNCMAP database to predict that SPI1 and ST8SIA1 were probable targets of NR2F1-AS1. SPI1 therein is a hematopoietic lineage-specific transcription factor belonging to the ETS-proto-oncogene, transcription factor family [24]. A previous study discovered that lncRNA small nucleolar RNA host gene 16 upregulated poly(ADP-ribose) polymerase family member 9 expression by recruiting the transcription factor, SPI1, to promote the tumorigenicity of cervical cancer cells [25]. Besides, ST8SIA1 was found down-regulated in pancreatic and liver cancer, and could inhibit these two types of tumor progression [26]. And ST8SIA1 knockout completely blocks the growth and metastasis of triple negative breast cancer cells in *vitro* and in *vivo* [27]. Another study suggested that microRNA-33a and let-7E inhibited the progression of colorectal cancer by targeting ST8SIA1 [28]. And ST8SIA1 was significantly enhanced in melanoma brain metastasis, and ST8SIA1 expression increased the levels of ganglioside GD3 on the cell surface [29]. The results of the present study revealed that NR2F1-AS1 could upregulate the expression levels of ST8SIA1 by recruiting SPI1 (Figure 6). Notably, the knockdown of NR2F1-AS1 expression suppressed the proliferation, invasion and migration of GC cells by regulating ST8SIA1.

However, there are limitations to the present study which should not be overlooked. First, the current study did not conduct experiments on more cell lines or clinical studies to further validate the significance of the NR2F1-AS1/SPI1/ST8SIA1 axis in GC progression. In addition, the diagnosis and treatment of GC is far more complicated than first anticipated. Therefore, it is important to further identify additional novel lncRNAs and mRNAs that may play a role in GC, and to determine their involvement in GC to help devise the most effective treatment strategy for GC.

## Conclusion

In conclusion, the findings of the present study indicated that NR2F1-AS1 may promote the proliferation, invasion and migration of GC cells by recruiting the transcriptional factor SPI1 to upregulate ST8SIA1 expression. Thus, regulating the expression of these factors may provide a novel strategy for the treatment of GC.

## Data Availability

The data that support the findings of this study are available from the corresponding author upon reasonable request.
